# Outcomes of initial antiretroviral treatment (ART) among recently diagnosed HIV patients in HIV-TR cohort, 2011–2012

**DOI:** 10.7448/IAS.17.4.19678

**Published:** 2014-11-02

**Authors:** Volkan Korten, Deniz Gökengin, Muzaffer Fincanci, Taner Yildirmak, Nuray Uzun Kes, Nuriye Taşdelen Fişgin, Dilara Inan, Haluk Eraksoy, Halis Akalin, Figen Kaptan

**Affiliations:** 1 Infectious Diseases, Marmara University Hospital, Istanbul, Turkey; 2 Infectious Diseases, Ege University Hospital, Izmir, Turkey; 3 Infectious Diseases, Istanbul Education and Research Hospital, Istanbul, Turkey; 4 Infectious Diseases, OkmeydanI Education and Research Hospital, Istanbul, Turkey; 5 Infectious Diseases, Sişli Education and Research Hospital, Istanbul, Turkey; 6 Infectious Diseases, MayIs University Hospital, Samsun, Turkey; 7 Infectious Diseases, Akdeniz University Hospital, Antalya, Turkey; 8 Infectious Diseases, Istanbul Medical School, Istanbul University, Istanbul, Turkey; 9 Infectious Diseases, Uludağ University Hospital, Bursa, Turkey; 10 Infectious Diseases, Izmir Education Training and Research Hospital, Izmir, Turkey

## Abstract

**Introduction:**

HIV-TR is a recently established (2012) multicentre cohort in Turkey. The aim of this study is to analyze epidemiological, immunologic and virologic data of recently diagnosed HIV patients.

**Materials and Methods:**

Epidemiologic, clinical and laboratory data of all patients diagnosed in 2011 and 2012 were recorded by a web-based data collection system, retrospectively.

**Results:**

A total of 693 patients (561 male, 132 female) at 24 sites were enrolled. The median age at first presentation for HIV care was 36. The proportion of patients presenting with advanced HIV disease (CD4 count<200/mm^3^ or presenting with an AIDS-defining event) was 30.6%; and 52.4% of patients were late presenters (CD4 count<350/mm^3^ or presenting with an AIDS-defining event). Median CD4 counts at presentation and before treatment were 344 (IQR: 175–540) and 295 (IQR: 150–430), respectively. Pretreatment CD4 count was >500 copies/mL in 18.5% of patients. Of 531 patients receiving ART, initial combinations consist of tenofovir/emtricitabine (TDF/FTC) plus efavirenz (EFV) in 48.2% and TDF/FTC plus lopinavir/ritonavir (LPV/r) in 37.5% and other combinations in 14.3% of the patients. Pre-treatment HIV-RNA was over 100.000 copies/mL in 52.3% of patients. At Weeks 24 and 48, HIV-RNA were<50 copies/mL in 63,4% of 385 patients and 82% of 311 patients reported to be still on ART and had a viral load measurement, respectively. Median pretreatment CD4 count was lower for TDF/FTC+LPV/r recipients than TDF/FTC+EFV recipients (250 vs 316) (p<0.05). The median increase from baseline CD4 cell count was 230 in TDF/FTC+LPV/r group, 193 in TDF/FTC+EFV group and 216 among all treated patients. Of 531 patients receiving ART, 11 had died and 19 were lost to follow-up.

**Conclusion:**

Despite 52.4% of recently diagnosed patients were late presenters; a high rate of virologic suppression was achieved in HIV-TR Cohort. A national HIV testing strategy targeting subpopulations with higher risk is urgently needed.

